# OsWHY1 Interacts with OsTRX z and is Essential for Early Chloroplast Development in Rice

**DOI:** 10.1186/s12284-022-00596-y

**Published:** 2022-10-08

**Authors:** Zhennan Qiu, Dongdong Chen, Linhong Teng, Peiyan Guan, Guoping Yu, Peiliang Zhang, Jian Song, Qiangcheng Zeng, Li Zhu

**Affiliations:** 1 Shandong Key Laboratory of Functional Biological Resources Development and Utilization in Universities, College of Life Science, Dezhou University, Dezhou, 253023 China; 2 State Key Laboratory of Rice Biology, China National Rice Research Institute, Hangzhou, 310006 China; 3 National Nanfan Research Institute, Chinese Academy of Agricultural Sciences, Sanya, 572000 China

**Keywords:** OsWHY1, OsTRX z, Chloroplast development, Rice

## Abstract

WHIRLY (WHY) family proteins, a small family of single-stranded DNA (ssDNA) binding proteins, are widely found in plants and have multiple functions to regulate plant growth and development. However, WHY in rice has received less attention. In this study, we continued our previous study on OsTRX z that is important for chloroplast development. OsTRX z was discovered to interact with OsWHY1, which was confirmed using yeast two-hybrid, pull-down, and BiFC assays. Subsequently, the *oswhy1* mutants were obtained by CRISPR/Cas9, which exhibited an albino phenotype and died after the three-leaf stage. Consistent with this albino phenotype, low amounts of Chl *a*, Chl *b,* and Car were detected in the *oswhy1-1* mutant. Moreover, the *oswhy1-1* mutant had chloroplasts with disrupted architecture and no stacked grana and thylakoid membranes. Subcellular localization showed that the OsWHY1-GFP fusion protein was targeted to the chloroplast. What’s more, *OsWHY1* was found to be preferentially expressed in young leaves and was involved in chloroplast RNA editing and splicing. Mutation of *OsWHY1* significantly affected the expression of chloroplast and ribosome development-related and chlorophyll synthesis-related genes. In conclusion, OsWHY1 contributes to early chloroplast development and normal seedling survival in rice. These results will further elucidate the molecular mechanism of chloroplast development and expand our understanding of WHY1 functions.

**Supplementary Information:**

The online version contains supplementary material available at 10.1186/s12284-022-00596-y.

## Background

It is well documented that the transcription of chloroplast genes depends on the nucleus-encoded RNA polymerase (NEP) and the plastid-encoded RNA polymerase (PEP) (Hedtke et al. 1997; Shiina et al. 2005). Thioredoxin z (TRX z) is a component of PEP and plays an important role in chloroplast development (Arsova et al. 2010; Lv et al. 2017; He et al. 2018). TRX z interacts with the fructokinase-like proteins FLN1 and FLN2, which are identified as components of the plastid transcriptionally active chromosome (pTAC) and contribute to PEP activity (Arsova et al. 2010; Lv et al. 2017; He et al. 2018). TRX z also interacts with other proteins related to chloroplast development, such as the CHLI subunit of Mg-chelatase, putative plastidic oxidoreductase TSV, Arabidopsis PLASTID REDOX INSENSITIVE2 (PRIN2), Fe-superoxide dismutase ALM1, and plastid multiple organellar RNA editing factors (OsMORFs) (Sun et al. 2017; Diaz et al. 2018; Wang et al. 2021a, 2021b). In brief, TRX z is essential for chloroplast development, but further research is needed to better understand its function and regulatory network.

WHIRLY (WHY) proteins are a small family of single-stranded DNA (ssDNA) binding proteins. The residues in the WHY domain (KGKAAL, YDW, and K) are conserved and are important for ssDNA activity (Desveaux et al. 2005). In monocotyledonous plants, the WHY family has two members (WHY1 and WHY2), whereas there are three members (WHY1, WHY2, and WHY3) in dicotyledonous plants (Desveaux et al. 2005). WHY family proteins can be found in the nucleus, plastids, or mitochondria (Cappadocia et al. 2010). In Arabidopsis, WHY1 is found in both the nucleus and the chloroplasts, WHY2 is found in the mitochondria, and WHY3 is found in the chloroplasts (Krause et al. 2005; Ren et al. 2017). Furthermore, WHY1 and WHY3 have been identified in the proteome of the transcriptionally active chromosome, which is the transcriptionally active fraction of the nucleoids that consists of multiple copies of ptDNA, RNA, and many proteins (Pfalz et al. 2006; Krupinska et al. 2014). Dual localization of WHY1 in the nucleus and the chloroplasts was also detected in barley and tomato (Grabowski et al. 2008; Zhuang et al. 2019).

WHY family proteins are widely found in plants and have multiple functions to regulate plant growth and development (Prikryl et al. 2008; Yan et al. 2020). Potato (*Solanum tuberosum*) WHY1 proteins were initially identified as transcriptional activators binding to the elicitor response element (ERE, TGACAnnnnTGCA) in the promoter region of the pathogenesis-related gene *PR-10a* (Despres et al. 1995; Desveaux et al. 2005). Knockout of *ZmWHY1* causes albino plants that lack plastid ribosomes and alter the splicing of *atpF*, indicating *ZmWHY1* plays an essential role in the biogenesis of chloroplasts in maize (*Zea mays*) (Prikryl et al. 2008). In barley (*Hordeum vulgare*), WHY1 protein in the nucleus can regulate the expression of senescence-associated gene *hvS40* by binding to the promoter of *hvS40*, whereas WHY1 protein in the chloroplasts is associated with intron-containing RNA processing (Melonek et al. 2010; Krupinska et al. 2014). The tomato (*Solanum lycopersicum*) WHY1 protein can bind to the upstream region of *psbA* in chloroplasts and regulate the expression of genes related to starch synthesis and degradation in the nucleus, which enhances the resistance of the tomato to chilling stress (Zhuang et al. 2019). Furthermore, MeWHYs can interact with MeCIPK23 to promote abscisic acid biosynthesis and regulate drought resistance in cassava (*Manihot esculenta*) (Yan et al. 2020).

In Arabidopsis, there are three genes coding for WHY proteins (WHY1, WHY2, and WHY3). The Arabidopsis mutant *why1* has no obvious phenotype (Marechal et al. 2009). The *why1why3* mutant has variegated green/white/yellow leaves in 5% of the progeny and is largely phenotypically similar to the wild-type (Marechal et al. 2009; Cappadocia et al. 2010). However, the triple mutant *why1why3pol1b-1*, which is deficient in WHY1, WHY3, and the chloroplast DNA polymerase 1 B (Pol1B), has a severe yellow-variegated phenotype, increased DNA rearrangements, lower photosynthetic electron transport efficiencies, and a higher accumulation of reactive oxygen species in the chloroplast (Lepage et al. 2013). These results indicate that plastid WHY1 and WHY3 as well as POL1B act as safeguards against plastid genome instability in Arabidopsis. Moreover, WHY1 protein is suggested as a thioredoxin target, while WHY3 protein is regarded as a redox-affected protein in chloroplasts (Foyer et al. 2014). Similar to StWHY1, AtWHY1 can bind to the upstream of WRKY53 in a developmental stage-dependent manner during leaf senescence (Miao et al. 2013). Further studies showed that Calcineurin B-like-Interacting Protein Kinase14 (CIPK14) can phosphorylate WHY1 in the nucleus, and then the phosphorylated WHY1 regulates the expression of WRKY53 (Ren et al. 2017; Guan et al. 2018). In addition, it was verified that AtWHY1 is involved in light adaptation by interacting with light-harvesting protein complex I (LHCA1) and affecting the expression of genes related to photosystem I (PS I) and light-harvesting complexes (LHCIs) (Huang et al. 2017).

In the present study, continuing our previous work on OsTRX z, we found it interacted with OsWHY1, which was verified by yeast two-hybrid, pull-down, and bimolecular fluorescence complementation (BiFC) assays. The rice mutant *oswhy1* was obtained using the CRISPR/Cas9 system and showed an albino and seedling lethality phenotype. We found that OsWHY1 was localized to the chloroplast and involved in chloroplast RNA editing and splicing. Furthermore, *OsWHY1* affected the expression of chloroplast and ribosome development-related and chlorophyll synthesis-related genes. These results indicate that OsWHY1 plays a vital role in the early chloroplast development and normal seedling survival in rice.

## Results

### OsWHY1 Interacts with OsTRX z In Vitro and In Vivo

Some studies have suggested that thioredoxin z (TRX z) is identified as a component of the plastid transcriptionally active chromosome (pTAC) and contributes to PEP activity (Arsova et al. 2010; Lv et al. 2017; He et al. 2018). Based on our previous studies, OsTRX z is located in chloroplasts, and knockout of *OsTRX z* causes albino and seedling lethality phenotypes, indicating that it plays an important role in chloroplast development (He et al. 2018). The plastid-located WHY1 is also identified as a pTAC and suggested as a thioredoxin target (Pfalz et al. 2006; Foyer et al. 2014). Furthermore, homozygous Z*mwhy1-1* mutants exhibit an albino seedling phenotype and die after the development of three to four leaves, which is similar to the phenotype of the rice mutant *ostrx z* (Prikryl et al. 2008; He et al. 2018). Based on the aforementioned experimental results, we hypothesized that OsTRX z would interact with OsWHY1 in rice to further explore the regulatory network of OsTRX z. According to the Rice Genome Annotation Project (RGAP) annotation and National Center for Biotechnology Information (NCBI), *LOC_Os06g05350* encodes the WHY1 protein that has a whirly structural domain and is a member of the whirly superfamily, so it was dubbed OsWHY1.

The *OsWHY1* is made up of six exons and five introns. The coding sequence of *OsWHY1* is 819 bp and encodes a polypeptide of 273 amino acids. Phylogenetic analysis was conducted to study the evolutionary relationship among WHY1 proteins. There were distinct monocotyledonous and dicotyledonous subdivisions, and the OsWHY1 was closely related to the grass family containing *maize*, *sorghum*, and *panicum* (Additional file 1: Fig. S1a). Furthermore, protein alignment indicated that OsWHY1 was highly conserved in higher plants, and it exhibited the highest similarity to the orthologs in *Setaria italica* (82%), *Brachypodium distachyon* (80.66%), and *Zea mays* (76%) (Additional file 1: Fig. S1b).

**Fig. 1:**
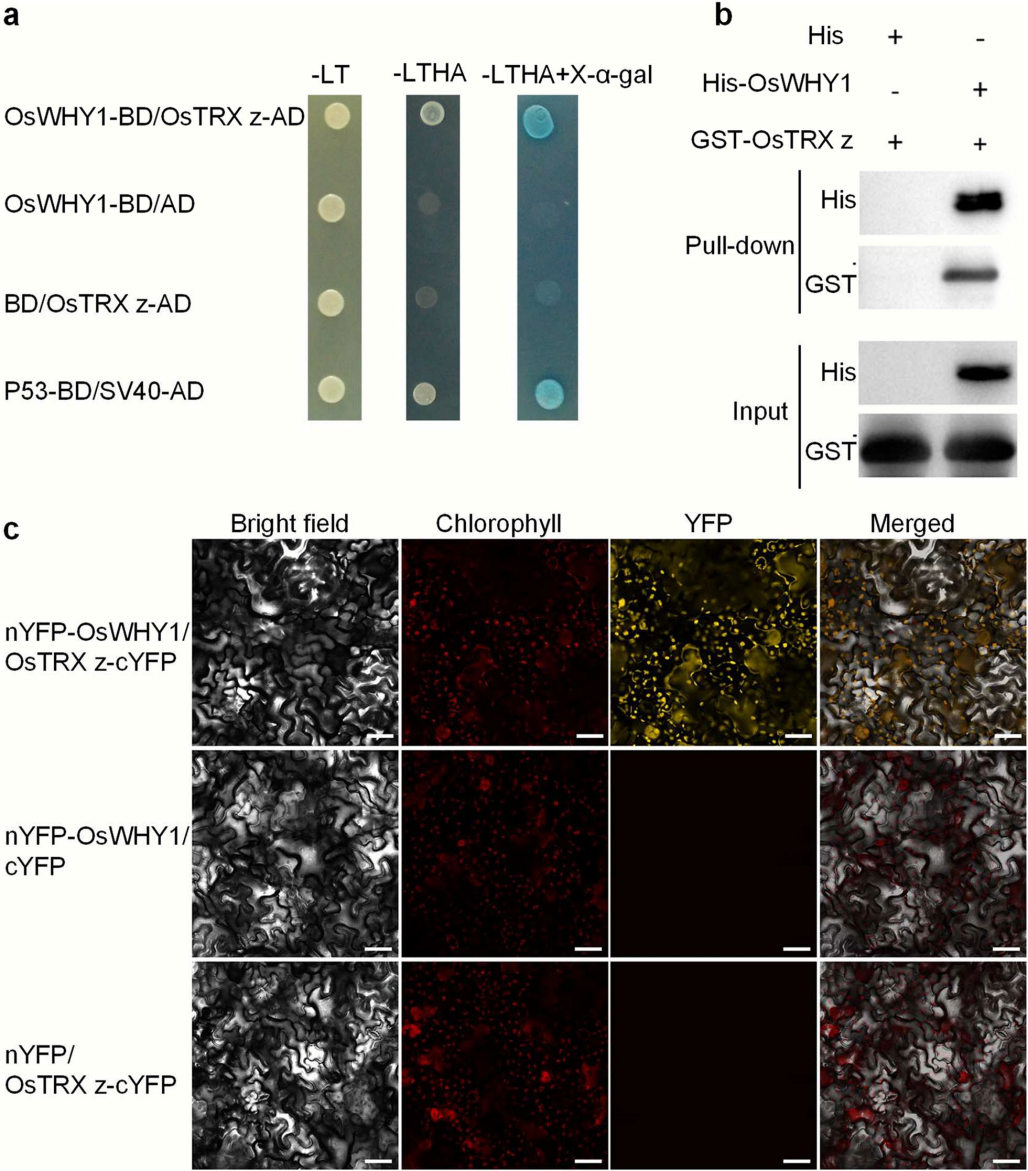
OsWHY1 interacts with OsTRX z in vitro and in vivo*.* **a** Interaction of OsWHY1 and OsTRX z in yeast two-hybrid assays. -LT: selective medium (SD/-Leu/-Trp); -LTHA: selective medium (SD/-Leu/-Trp/-His/-Ade); -LTHA + X-α-gal: selective medium (SD/-Leu/-Trp/-His/-Ade) containing X-α-gal. P53-BD and SV40-AD were used as positive controls. **b** Pull-down assays for detection of interaction of His-OsWHY1 and GST-OsTRX z in vitro. **c** Bimolecular fluorescence complementation (BiFC) assays demonstrating that OsWHY1 interacts with OsTRX z in *Nicotiana benthamiana* leaf cell chloroplasts. Bar = 50 μm

To confirm the prediction that OsWHY1 interacts with OsTRX z, we performed yeast two-hybrid (Y2H) assays to verify. Y2H assays revealed that OsWHY1 interacted with OsTRX z (Fig. 1a). In addition, pull-down assays confirmed the interaction in vitro (Fig. 1b). The interaction of OsWHY1 with OsTRX z in the chloroplast of *N.benthamiana* leaf cells was verified in vivo using BiFC assays (Fig. 1c). When nYFP-OsWHY1 and OsTRX z-cYFP combined, there was strong YFP fluorescence with a punctate localization pattern in the chloroplast (Fig. 1c). These results strongly suggest that OsWHY1 interacts with OsTRX z in vitro and in vivo.

### Loss-of-Function *oswhy1* Mutants Exhibit an Albino-Lethal Phenotype at the Seedling Stage

**Fig. 2:**
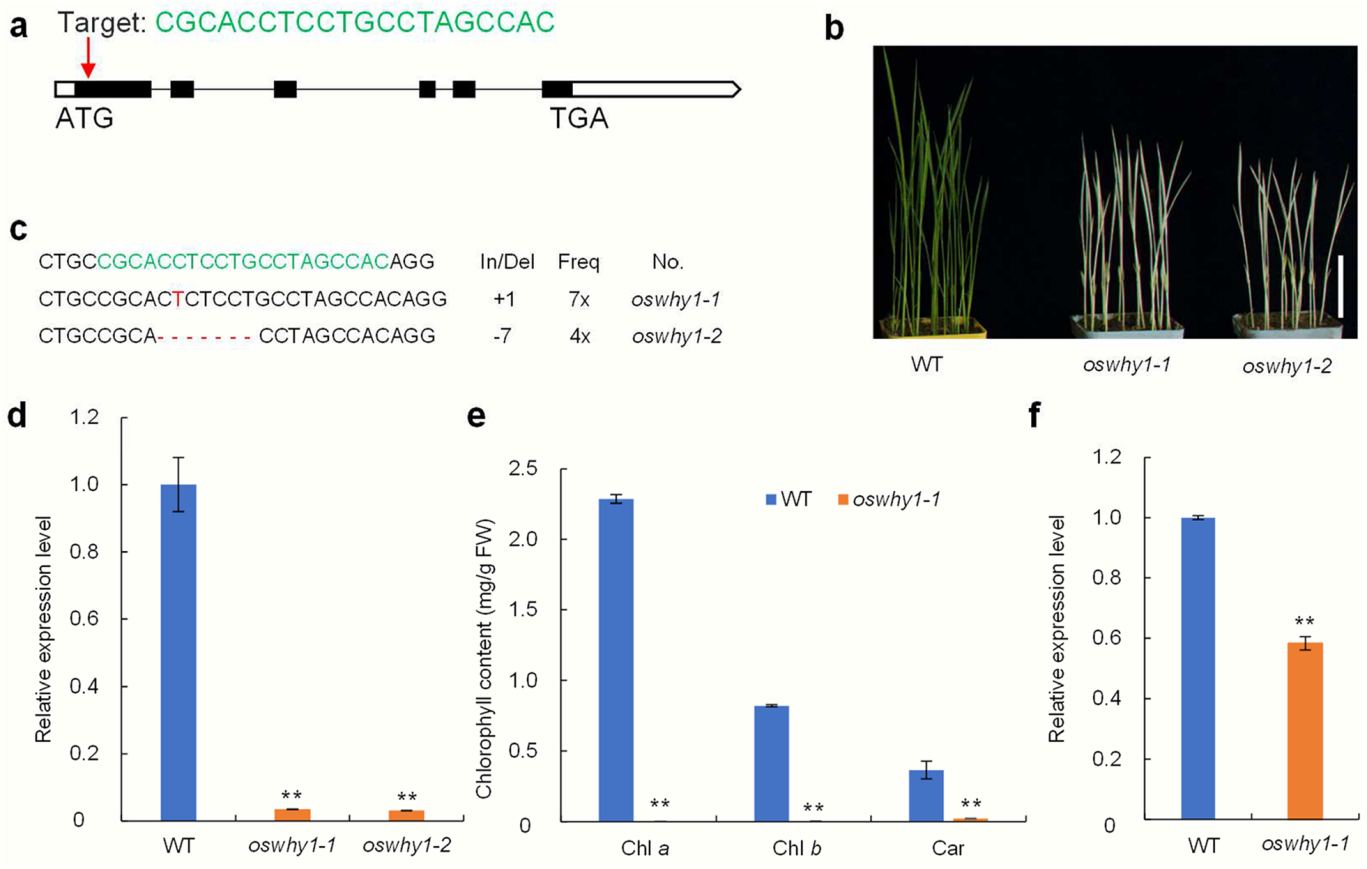
Disruption of *OsWHY1* function causes an albino-lethal phenotype at the seedling stage. **a** Schematic diagram showing the target site of *OsWHY1* by the CRISPR/Cas9 system. The target sequences within the first exon of *OsWHY1* are marked in green. **b** Phenotypes of the wild-type (WT) and the *oswhy1* mutant at the two-leaf stage. Seedlings were grown in a growth chamber with 14 h of light (300 μmol photons m^−2^ s^−1^) and 10 h of darkness at constant temperatures of 30 °C. Bar = 3 cm. **c** Two modes of mutation in the *oswhy1* mutants. The *oswhy1-1* mutant is an insertion mutation, and the *oswhy1-2* is a deletion mutation. **d** Relative *OsWHY1* transcript levels in the WT and *oswhy1* plants. The *OsWHY1* transcript levels in the WT were set to 1. **e** The contents of chlorophyll *a* (Chl *a*), chlorophyll *b* (Chl *b*), and carotenoid (Car) in the WT and *oswhy1-1* plants at the two-leaf stage. f Relative *OsTRX z* transcript levels in the WT and *oswhy1-1*. Data are the mean ± SD (n = 3). **p < 0.01 (Student’s *t*-test)

To investigate the function of OsWHY1 in rice, we chose a target site and used a CRISPR/Cas9-mediated genome editing system to create the pWHY1-cas9 expression vector (Fig. 2a). The pWHY1-cas9 expression vector was introduced into *japonica cultivar* Zhonghua11 (ZH11) through an *Agrobacterium*-mediated transformation. Sequence analysis revealed that there were 11 independent T_0_ generation transgenic lines that exhibited heterozygous mutations. According to phenotypic observations, all *oswhy1* mutants derived from the T_1_ generation of *oswhy1*/*OsWHY1* heterozygous plants showed an albino phenotype and died after the three-leaf stage, and we sequenced the target site of the albino mutants and discovered two mutation patterns (1 bp insertion and 7 bp deletion), both of which caused premature stop codons (Fig. 2b, c). Subsequently, we extracted RNA and analyzed *OsWHY1* expression levels in the WT and *oswhy1* mutants by quantitative real-time PCR (qRT-PCR). The results revealed that the expression of *OsWHY1* was significantly reduced in the *oswhy1-1* and *oswhy1-2* mutants compared to the WT (Fig. 2d). Since both the *oswhy1-1* and *oswhy1-2* mutants are nonsense mutations with the same phenotype, we selected one of them, *oswhy1-1*, to be used as a representative line in the following experiments. Low levels of Chl *a*, Chl *b*, and Car were found in the *oswhy1-1* mutant, which is consistent with the albino phenotype (Fig. 2e). Given that OsWHY1 was discovered to interact with OsTRX z, we examined the expression of *OsTRX z* in the *oswhy1-1* mutant and WT. The qRT-PCR results showed that the *OsTRX z* expression was remarkably suppressed in the *oswhy1-1* mutant compared to the WT, suggesting that the function of OsTRX z is impaired due to the mutation of OsWHY1 (Fig. 2f). According to previous studies, many rice albino mutants show re-greening at high or low temperatures, such as *tsv*, *hsa1,* and *osv4* (Gong et al. 2014; Sun et al. 2017; Qiu et al. 2018a). To determine whether the *oswhy1-1* mutant is sensitive to temperature, we performed different temperature treatment experiments. We found that temperature had no effect on the albino-lethal phenotype of the *oswhy1-1* mutant, and its phenotype remained constant at 30 °C and 20 °C (Additional file 1: Fig. S2). These findings demonstrate that WHY1 is essential for rice growth and development, and its effect on phenotype is not controlled by temperature.

### Chloroplast Development is Affected in the *oswhy1-1* Mutant

**Fig. 3:**
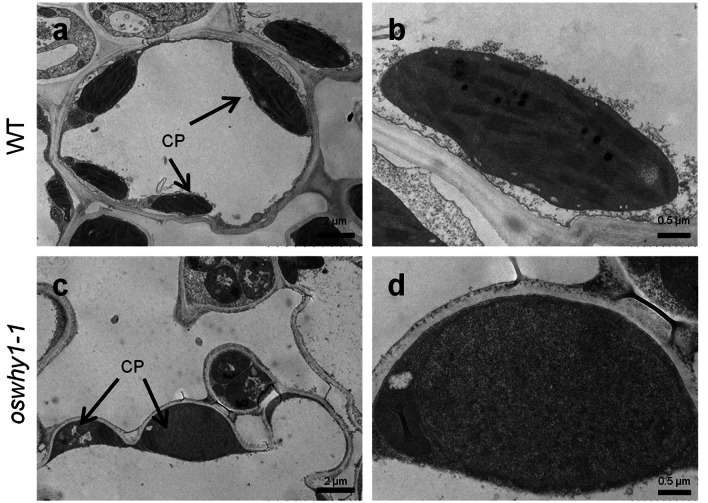
Transmission electron micrograph (TEM) of chloroplasts in the WT and *oswhy1-1* leaves. Chloroplast structures in leaves of the WT (**a**, **b**) and *oswhy1-1* (**c**, **d**) at the seedling stage. CP, chloroplast. **b** and **d** were the enlarged images of **a** and **c**. CP, chloroplast. Bar (**a**, **b**) = 2 μm; Bar (**c**, **d**) = 0.5 μm

To determine whether chloroplast development is affected by the *OsWHY1* mutation, we analyzed the chloroplast ultrastructure of WT and *oswhy1-1* seedlings at the three-leaf stage with transmission electron microscopy. The chloroplasts isolated from the WT leaves were well-structured, with densely arranged grana lamellae (Fig. 3a, b). However, the *oswhy1-1* mutant had chloroplasts with disrupted architecture, as well as no stacked grana and thylakoid membranes (Fig. 3c, d). These results indicate that the mutation of *OsWHY1* impairs chloroplast development.

### Subcellular Localization and Expression Pattern Analysis

**Fig. 4:**
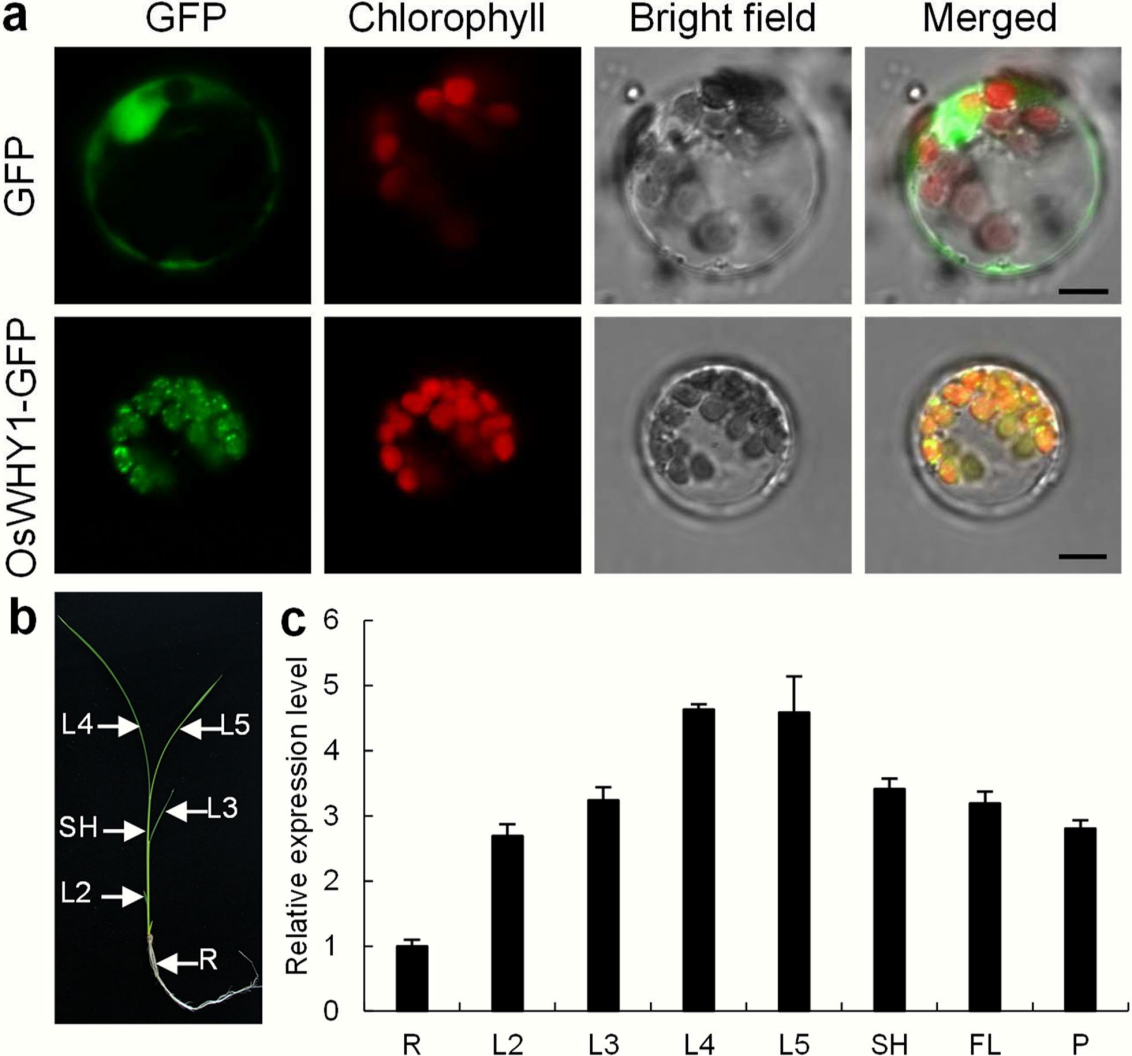
Subcellular localization and expression analysis. **a** Subcellular localization of OsWHY1. Free (GFP) and OsWHY1-GFP fusion protein in rice protoplasts were photographed under a confocal microscope at 488 nm. Bar = 5 μm. **b, c** *OsWHY1* expression levels in different tissues of the WT seedlings. L2-L5, the second to the fifth leaves; SH, leaf sheath; R, root; FL, flag leaf; P, panicle. The *OsWHY1* transcript levels in the root were set to 1. Data are the mean ± SD (n = 3). **p < 0.01 (Student’s *t*-test)

To explore the subcellular localization of OsWHY1, the pOsWHY1-GFP fusion vector was constructed and transformed into rice protoplasts, with an empty pGFP vector serving as a control. Confocal microscopy results revealed that the OsWHY1-GFP fusion protein produced signals that co-localized with the chloroplast autofluorescence signals, whereas the empty GFP protein was found in the cytoplasm and the nucleus (Fig. 4a). According to subcellular location prediction of PPDB (http://ppdb.tc.cornell.edu/dbsearch/searchacc.aspx), the N-terminal 50 amino acids are the chloroplast transit peptide (CTP) sequences of OsWHY1 (Additional file 1: Fig. S3a). Then, the CTP_OsWHY1_-GFP vector and ΔCTP_OsWHY1_-GFP vector were constructed and transformed into rice protoplasts. The results showed that the CTP_OsWHY1_-GFP fusion protein was located in the chloroplast (Additional file 1: Fig. S3b), and the ΔCTP_OsWHY1_-GFP fusion protein was located in the cytoplasm (Additional file 1: Fig. S3c). Further experiments showed the ΔCTP_OsWHY1_-GFP fusion protein was not merged with the Ghd7-mCherry (a nuclear localization marker) (Tu et al. 2020) (Additional file 1: Fig. S3c). These results suggest that the OsWHY1 N-terminals have a CTP that mediates the transport of OsWHY1 from the cytoplasm into the chloroplast. In addition, qRT-PCR was performed to further determine the expression pattern of *OsWHY1* in the WT plants. According to the qRT-PCR results, *OsWHY1* was preferentially expressed in young leaves (Fig. 4b, c). OsWHY1 appears to serve a function in early chloroplast synthesis in rice leaves during the seedling stage, as evidenced by higher expression in young leaves, chloroplast localization, chloroplast damage, and an albino-lethal phenotype.

### Mutation of *OsWHY1* Affects the Splicing of the Plastid-Encoded *ndhA* Transcripts

**Fig. 5:**
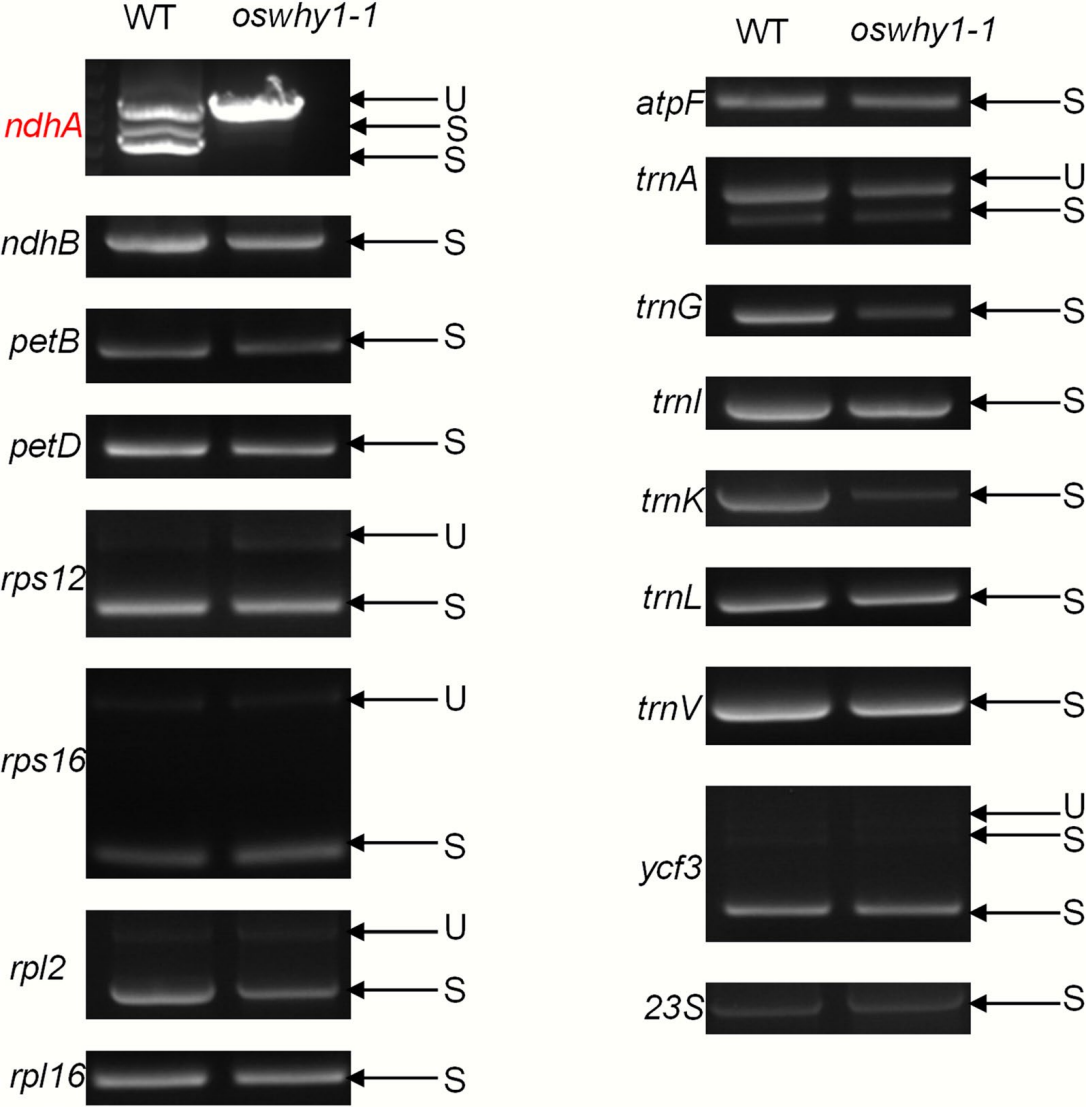
Splicing analysis of rice chloroplast genes in the WT and *oswhy1-1* leaves. U indicates unspliced transcripts; S indicates spliced transcripts

Chloroplast development is regulated by post-transcriptional regulation, including RNA processing, editing, and splicing, which further affects the transcript levels of chloroplast genes (Stern et al. 2010). Previous studies have shown that homozygous Zm*why1-1* mutants exhibit an albino seedling phenotype and die after the development of three to four leaves (Prikryl et al. 2008). Further research revealed that the Zm*why1-1* mutants’ chloroplast development is blocked, resulting from aberrant *atpF* intron splicing and a decrease in the content of plastid ribosomes (Prikryl et al. 2008). Based on the findings of ZmWHY1, we wonder whether OsWHY1 is likewise involved in the RNA splicing of chloroplast genes. The RNA splicing of chloroplast genes has one group-I intron and 17 group-II introns (Hiratsuka et al. 1989). We performed RT-PCR with primers flanking the introns and compared the lengths of the amplified products between the WT and the *oswhy1-1* mutant. One chloroplast transcript, *ndhA*, was severely suppressed in the splicing of *oswhy1-1* compared to WT, and the splicing of *atpF* in rice was not affected by the OsWHY1 mutation (Fig. 5). These results suggest that OsWHY1 is specifically required for the splicing of the group-II *ndhA* plastid transcripts in rice, which is different from the function of ZmWHY1 for chloroplast *atpF* splicing.

### OsWHY1 Participates in Chloroplast RNA Editing

**Fig. 6:**
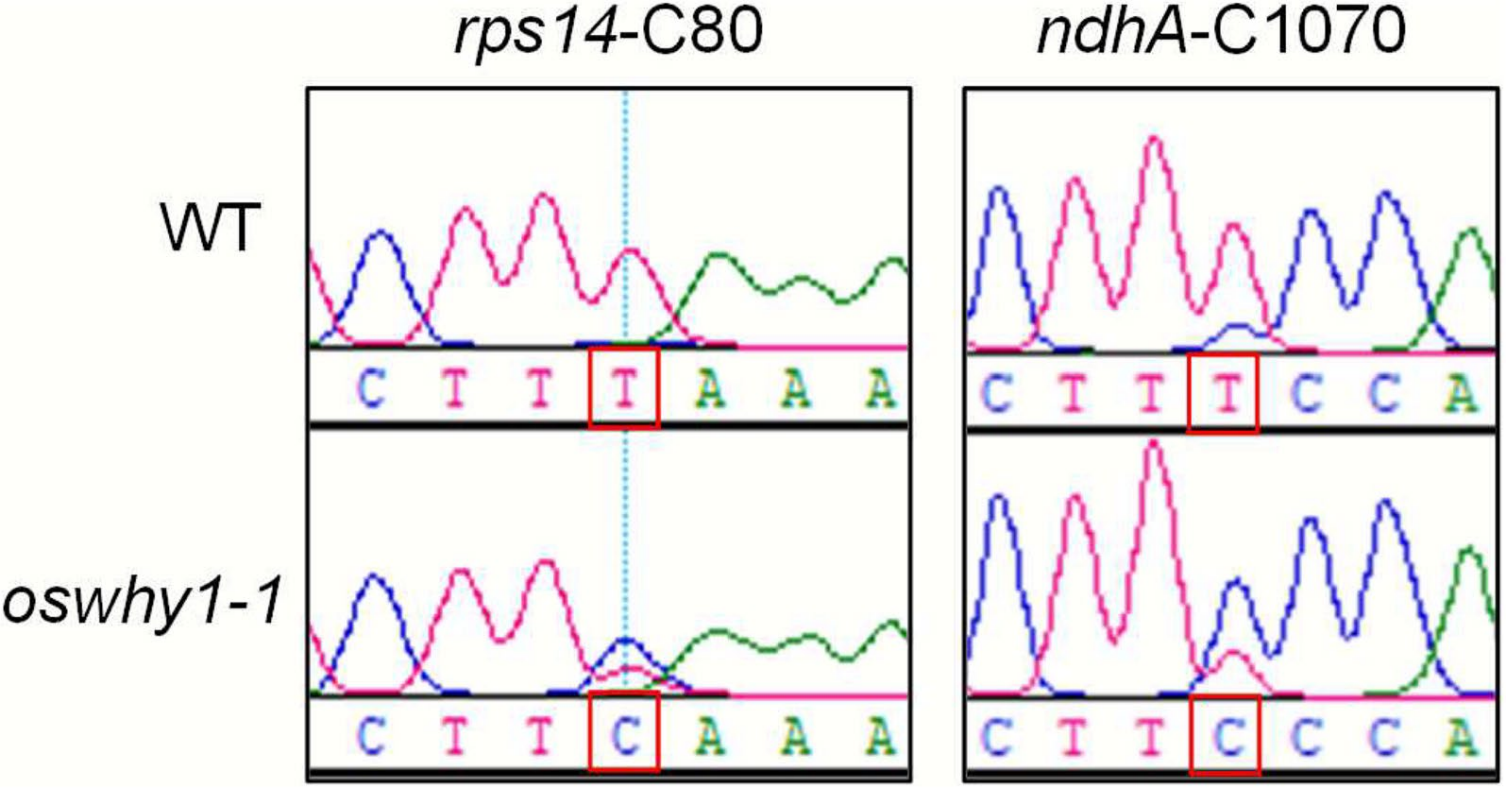
OsWHY1 affects chloroplast RNA editing sites *ndhA* and *rps14.* Two plastid genes and the corresponding two editing sites were not normally edited in the *oswhy1-1* mutant. The red boxes indicate RNA editing sites (T) in the WT and non-editing sites (C) in the *oswhy1-1*

It was proven that OsTRX z is implicated in chloroplast RNA editing (Wang et al. 2021b). Considering the interactions between OsWHY1 and OsTRX z, we tested whether OsWHY1 affects RNA editing at chloroplast sites. RNA was extracted from the WT and *oswhy1-1* plants at the three-leaf stage. The 23 known chloroplast RNA editing sites were sequenced in the WT and *oswhy1*-*1* plants. The sequence analysis results showed that the majority of the editing sites were routinely edited in the *oswhy1-1* mutant compared to the WT, except for *ndhA*-C1070 and *rps14*-C80 (Fig. 6 and Additional file 2: Table S1). These results indicate that OsWHY1 is essential for chloroplast RNA editing. It is also worth noting that OsWHY1 facilitates the *ndhA* splicing and editing.

### The Expression Levels of Genes Related to Chloroplast Development, Chlorophyll Synthesis, and Ribosome Development are Altered in the *oswhy1-1* Mutant

**Fig. 7:**
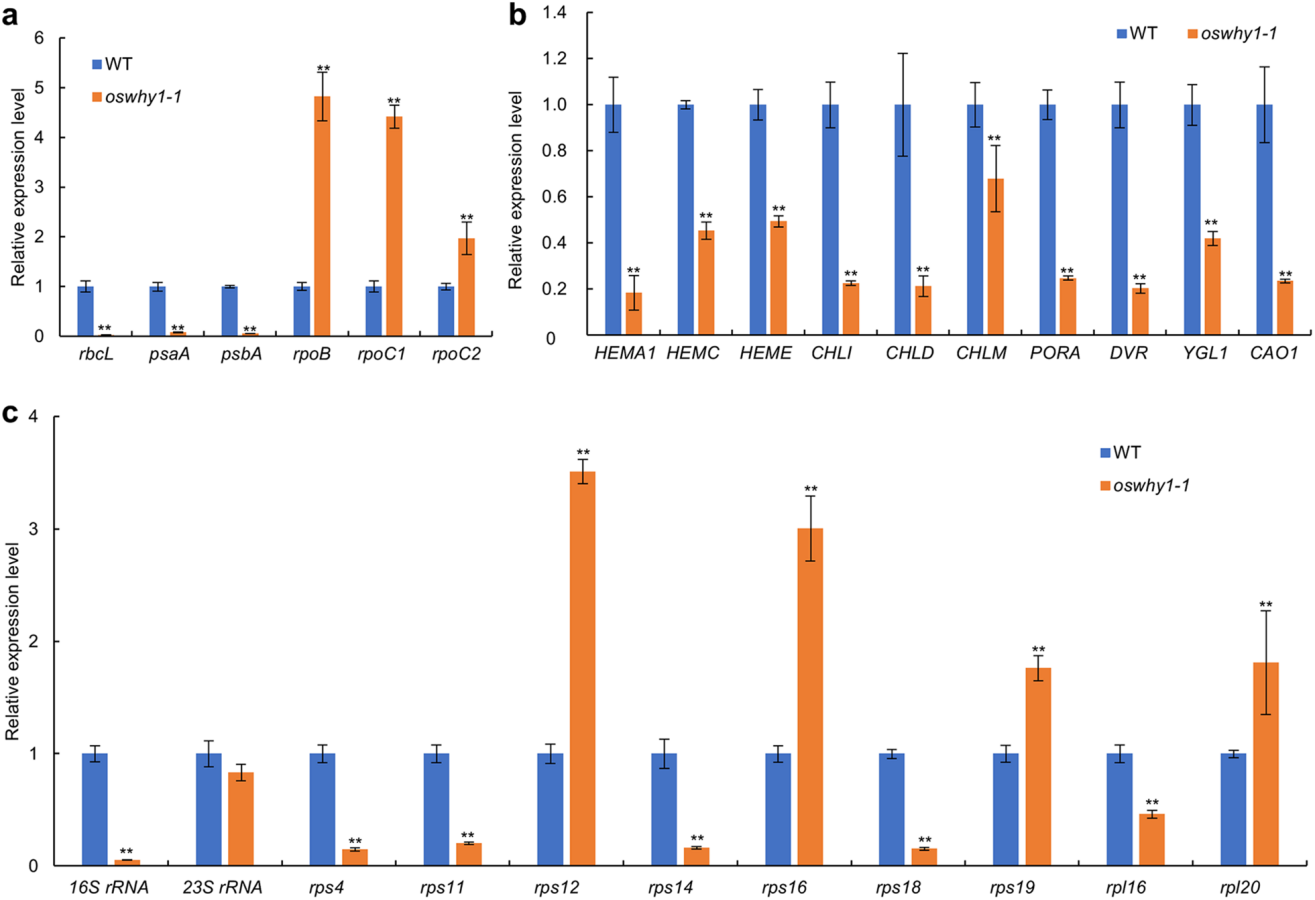
Analysis of the relative transcript levels of genes related to chloroplast development, chlorophyll synthesis, and ribosome development. **a** The relative expression levels of plastid-encoded and nucleus-encoded chloroplast development-related genes. **b** Transcript analysis of chlorophyll synthesis-related genes. **c** The relative expression levels of genes associated with ribosome development. The relative expression level of each gene was normalized using *UBQ5* as an internal control. The expression level of each gene in the WT was set to 1.0 and the other samples were calculated accordingly. Data are the mean ± SD (n = 3). **p < 0.01 (Student’s *t*-test)

The WHY1 protein is a component of the pTAC complex, which affects PEP activity and chloroplast development (Pfalz et al. 2006). Furthermore, OsTRX z is essential for PEP activity and regulates the expression of PEP-related genes during chloroplast development (He et al. 2018; Wang et al. 2021b). To investigate whether chloroplast genes are altered in the *oswhy1-1* mutants, the qRT-PCR assay was performed to examine the expression of NEP-dependent genes (*rpoB*, *rpoC1*, and *rpoC2*) and PEP-dependent genes (*psaA*, *psbA*, and *rbcL*). The expression levels of PEP-dependent genes were lower in the *oswhy1-1* mutant than in the WT (Fig. 7a). In the *oswhy1-1* mutant, the transcript levels of NEP-dependent genes containing *rpoB*, *rpoC1*, and *rpoC2* were dramatically elevated (Fig. 7a). The chlorophyll synthesis genes *HEMA1*, *HEMC*, *HEME*, *CHLI*, *CHLD*, *CHLM*, *PORA*, *DVR*, *YGL1*, and *CAO1* were severely suppressed in the *oswhy1-1* mutant (Fig. 7b). Consequently, the *oswhy1-1* mutant had lower Chl *a*, Chl *b*, and Car content than the WT. In addition, the expression levels of genes associated with ribosome development were also detected. *16S rRNA*, *rps4*, *rps11*, *rps14*, *rps18*, and *rpl16* were all found to be significantly reduced in the *oswhy1-1* mutant compared to the WT, with *16S rRNA* almost undetectable. Nonetheless, in the *oswhy1-1* mutant, *rps12*, *rps16*, *rps19*, and *rpl20* were up-regulated (Fig. 7c). Based on these findings, OsWHY1 plays an important role in the transcript expression of genes involved in chloroplast and ribosome development as well as chlorophyll synthesis.

## Discussion

WHY proteins have been widely studied in Arabidopsis, potato, maize, barley, cassava, and tomato, whereas rice has received less attention. In this study, we identified a rice *OsWHY1* gene, mutation of which resulted in an albino and seedling lethality phenotype (Fig. 2b). Consistent with the albino phenotype, the *oswhy1*-*1* mutant had low amounts of Chl *a*, Chl *b*, and Car (Fig. 2e). Compared to the WT, there were few and undifferentiated chloroplasts with no thylakoid membranes in the *oswhy1-1* leaves (Fig. 3). The OsWHY1-GFP fusion protein was targeted to the chloroplast (Fig. 4a). Moreover, *OsWHY1* was preferentially expressed in young leaves (Fig. 4c). The above results indicate that OsWHY1 plays a significant role in chloroplast development in rice leaves at the seedling stage.

WHY1 belongs to a single-stranded DNA/RNA binding protein that can be localized in the nucleus and chloroplasts (Grabowski et al. 2008; Prikryl et al. 2008; Marechal et al. 2009). Previous studies have shown that the AtWHY1-GFP and HvWHY1-GFP fusion proteins are exclusively targeted to the plastids, and transient transformation experiments did not provide any evidence for localization in the nucleus (Krause et al. 2005; Melonek et al. 2010). Subsequent experiments revealed that AtWHY1 and HvWHY1 are detected in chloroplasts and nucleus of the same cell using immunoblot and immunohistochemical methods (Grabowski et al. 2008; Melonek et al. 2010; Ren et al. 2017). In the work reported here, subcellular localization showed that OsWHY1-GFP fusion protein was only targeted to the chloroplast (Fig. 4a), which is similar to the results of AtWHY1-GFP and HvWHY1-GFP. Further research indicated that the OsWHY1 N-terminals have a CTP that mediates the transport of OsWHY1 from the cytoplasm into the chloroplast (Additional file 1: Fig. S3). Why is the WHY1-GFP fluorescence not found in the nucleus? The main reason for this result may be the high molecular mass of the fusion protein (Krause et al. 2005; Melonek et al. 2010). WHY1 proteins are often tiny enough to reach the nucleus passively, whereas WHY1-GFP fusion proteins would require a nuclear localization signal to be imported into the nucleus (Krause et al. 2005). It is also possible that the majority of WHY1 is directed toward plastids, with so little remaining in the nucleus that GFP fluorescence misses it (Comadira et al. 2015). Further studies are needed to determine whether the subcellular localization of OsWHY1 is dual-localized. However, what is certain is that OsWHY1 is targeted to the chloroplast.

WHY1 deficiency affects different plant phenotypes in different ways. Knockout AtWHY1 mutants have no apparent phenotype, and the double-knockout mutants *why1why3* lacking WHY1 and WHY3 are largely phenotypically similar to the WT, whereas the triple-mutants *why1why3pol1b-1* (defective WHY1, WHY3, and POL1B) with high plastid genome instability and reactive oxygen species content show a distinct variegated phenotype (Marechal et al. 2009; Lepage et al. 2013). In maize (*Zea mays*), ZmWHY1 is found in the chloroplast, and the homozygous Zm*why1-1* mutants exhibit an albino seedling phenotype and die after the development of three to four leaves due to reduced *atpF* intron splicing and reduced content of plastid ribosomes (Prikryl et al. 2008). In barley, the RNA interference-knockdown lines with extremely low levels of *HvWHY1* transcripts are phenotypically similar to the WT (Melonek et al. 2010; Comadira et al. 2015). Knockdown of *HvWHY1* in transgenic barley also affects the splicing of *atpF* transcripts, while it has no impact on chloroplast development and genes coding for ribosomal RNAs or tRNAs (Melonek et al. 2010). Herein, the knockout of *OsWHY1* resulted in the same phenotype as the homozygous Zm*why1-1* mutants. However, our results showed that *OsWHY1* only affected the splicing of the plastid-encoded *ndhA* transcripts (Fig. 5), which was inconsistent with *ZmWHY1* and *HvWHY1*. From the above results, it appears that WHY1 can be involved in the splicing of chloroplast genes. Although both ZmWHY1 and HvWHY1 affect the splicing of *atpF* transcripts, they have different effects on their phenotypes. Mutations in *OsWHY1* and *ZmWHY1* cause similar albino-lethal phenotypes in rice and maize, but OsWHY1 affects the splicing of *ndhA* transcripts. The *ndhA* gene encodes a subunit of the chloroplast NADH dehydrogenase-like (NDH) complex. This *ndhA* splicing defect is also found in other rice leave color mutants, such as *wsp1*, *pgl12*, and *wsl4* (Wang et al. 2017; Zhang et al. 2017; Chen et al. 2019). The phenotypic changes in these mutants, however, are not attributed to the *ndhA* splicing defects but rather to other factors, such as impaired PEP activity and ribosome biosynthesis. In addition, knockout of the NDH complex in *Nicotiana tabacum* and *Physcomitrella patens* has no phenotype under normal conditions (Burrows et al. 1998; Ito et al. 2018). As a result, the *ndhA* gene may not be responsible for the phenotype of the *oswhy1-1* mutant, and there should be more significant explanations for the *oswhy1-1* mutant phenotype.

Several studies have demonstrated that the plastid-located WHY1 is identified as a plastid transcriptionally active chromosome (pTAC), and it has been indicated as a thioredoxin target (Pfalz et al. 2006; Foyer et al. 2014). According to our earlier research, OsTRX z is found in chloroplasts, and knocking it out causes an albino and seedling lethality phenotype (He et al. 2018). Our studies revealed that OsTRX z interacted with OsWHY1, which was verified by yeast two-hybrid, pull-down, and BiFC assays (Fig. 1). Moreover, the mutant *oswhy1* phenotype is identical to that of the mutant *ostrx z*. Given that OsTRX z regulates chloroplast RNA editing, we wonder whether OsWHY1 affects RNA editing. As a result, an RNA editing assay was performed. We observed defects in RNA editing of *rps14*-C80 and *ndhA*-C1070 in the *oswhy1-1* mutant (Fig. 6). Previous studies found that some rice albino mutants exhibit chloroplast RNA editing defects (Liu et al. 2021; Wang et al. 2021b). However, some studies indicate that the defective RNA editing cannot cause the albino phenotype. According to the studies of the rice mutants *wsl3*, *ostrx z,* and *osppr6*, there is no correlation between RNA editing defects and the albino phenotype (Tang et al. 2017; Wang et al. 2021b). Therefore, the editing defects in the *oswhy1-1* mutant are not responsible for the albino phenotype. It is necessary to mention that the plastid *ndhA* gene was not correctly spliced in the *oswhy1-1* mutant (Fig. 5). Based on these observations, we conclude that OsWHY1 is involved in chloroplast RNA editing and splicing but that this is not the main cause of the mutant phenotype.

During plant growth, chloroplasts play crucial roles in light reception and carbon sequestration. Chloroplast development is coordinately regulated by both plastid and nuclear genes, while nuclear genes control the majority of it due to the low coding capacity of plastids. (Yoo et al. 2009; Pfalz and Pfannschmidt 2013). NEP mainly transcribes plastid housekeeping genes, such as *rpoB*, *rpoC1*, and *rpoC2*, which are required during the early stage of chloroplast development. PEP then transcribes the photosynthetic genes like *psbA*, *psaA*, and *rbcL*. According to our results, the expression of PEP-dependent genes was relatively low, whereas the NEP-dependent genes were elevated to varying degrees in the *oswhy1-1* mutant (Fig. 7a), which is almost similar to the *ostrx z* mutant (He et al. 2018). The plastid-encoded gene expression variation revealed that PEP activity is inhibited in the *oswhy1-1* mutant. Because NEP preferentially transcribes plastid housekeeping genes, diminished translation of PEP genes in the chloroplast may activate NEP genes via feedback processes. Therefore, we conclude that the impairment of chloroplast development in the *oswhy1-1* mutant may be due to lower PEP activity that affects the transcription of chloroplast genes. Plastid ribosomal proteins (PRPs) play an important role in ribosome biogenesis, plastid protein biosynthesis, chloroplast differentiation, and early chloroplast development (Gong et al. 2013). Previous studies have shown that loss-of-function of genes that encode plastid ribosome can cause an albino phenotype and early seedling lethality in rice, such as *al1*, *asl1*, and *wgl2* (Gong et al. 2013; Zhao et al. 2016; Qiu et al. 2018b). We found that the transcript level of *16S rRNA* was dramatically reduced in the *oswhy1-1* mutant compared to the WT, whereas *23S rRNA* was less affected (Fig. 7c). Other genes related to ribosome development also displayed abnormal expression (Fig. 7c). The *16S rRNA* is necessary for mRNA binding and stability of codon-anticodon interaction. Thus, loss of function of OsWHY1 is assumed to affect the assembly and accumulation of plastid ribosomes, leading to disruption of plastid translation during chloroplast development. As a result of the impairment in plastid genes translation, there were no stacked grana and thylakoid membranes in the chloroplast of the *oswhy1-1* mutant (Fig. 3). In addition, the transcript levels of the chlorophyll synthesis genes were significantly reduced (Fig. 7b). Taken together, OsWHY1 affects the assembly and accumulation of plastid ribosomes and PEP activity, which may disturb the transcription and translation of chloroplast genes. Consequently, the impaired ribosome biosynthesis and PEP activity in the *oswhy1-1* mutant may account for the albino-lethal phenotype at the seedling stage.

## Conclusion

Plastid-located DNA/RNA binding protein OsWHY1 can interact with OsTRX z and affect chloroplast RNA editing and splicing, PEP activity, and plastid ribosomes development. In conclusion, OsWHY1 contributes to early chloroplast development and normal seedling survival in rice. These results will further elucidate the molecular mechanism of chloroplast development and expand our understanding of WHY1 functions.

## Materials and Methods

### Plant Materials and Growth Conditions

Using a CRISPR/Cas9 gene-editing technique, the *oswhy1*/*OsWHY1* heterozygous plants were obtained from the japonica rice cultivar ‘Zhonghua 11’. The gDNA target site (CGCACCTCCTGCCTAGCCACAGG) on the first exon of *OsWHY1* was used to create the pOsWHY1-Cas9 vector, which was transformed into the WT by *Agrobacterium*-mediated transformation, according to a previously described method (Qiu et al. 2018a). Seedlings were grown in a growth chamber with 14 h of light (300 μmol photons m^−2^ s^−1^) and 10 h of darkness at constant temperatures of 20 °C or 30 °C.

### Pigment Content Measurement

At the three-leaf stage, the leaf samples (0.1 g) of WT and *oswhy1-1* mutant were collected and cut into 1 cm segments and soaked in 10 ml 80% ethanol for 48 h at room temperature in the dark. The supernatant was measured at 662 nm (maximum absorption peak of Chl *a*), 646 nm (maximum absorption peak of Chl *b*), and 470 nm (maximum absorption peak of Car) light using a UV-1800PC spectrophotometer (Mapada, China). The concentrations of Chl *a*, Chl *b*, and Car were calculated using the methods of Arnon (1949) and Wellburn (1994) (Arnon 1949; Wellburn and Alan 1994). Three biological replicates were carried out.

### Transmission Electron Microscopy (TEM) Assays

At the three-leaf stage, the wild-type and *oswhy1-1* mutant leaf samples were cut into small pieces, fixed in 2.5% glutaraldehyde, and then in 1% OsO_4_. All samples were prepared for TEM using the methods described previously (Tan et al. 2014). The processed samples were examined using a Hitachi H-7650 electron microscope (Tokyo, Japan).

### RNA Extraction and Quantitative Real-Time PCR (qRT-PCR)

Total rice RNA was extracted and purified from different tissues using an RNAprep Pure Plant Kit (Tiangen Co., Beijing, China) according to the manufacturer’s protocols. The cDNA was reverse transcribed according to the manufacturer’s instructions using the ReverTraAce quantitative PCR RT Master Mix Kit with gDNA remover (Toyobo, Japan). The qRT-PCR was performed on the Light Cycle 96 System using the SYBR Green real-time PCR master mix (TOYORO, Japan). The rice *UBQ5* gene was used as an internal control. All primers for qRT-PCR are listed in Additional file 2: Table S2. The data were presented as the mean ± SD of three biological replicates. A Student’s *t*-test was used for statistical analysis.

### Chloroplast RNA Splicing and Editing Analysis

According to previous studies, RT-PCR was used to analyze the splicing of group I and group II introns of chloroplast genes from the WT and *oswhy1-1* mutants (Wang et al. 2017; Zhang et al. 2017). RT-PCR amplification with the appropriate primers yielded specific cDNA fragments, which were analyzed to identify C to T alterations caused by RNA editing (Wang et al. 2017; Zhang et al. 2017). All primers for chloroplast RNA splicing and editing analysis are listed in Additional file 2: Table S2.

### Subcellular Localization of OsWHY1 Protein

To explore the subcellular localization of OsWHY1, the full-length *OsWHY1* coding sequence without the termination codon with primers OsWHY1-GFPF/R (Additional file 2: Table S2) was amplified and cloned into the GFP vector using a ClonExpress ® II One Step Cloning Kit (Vazyme Biotech, Nanjing, China) (Qiu et al. 2018b). The pOsWHY1-GFP vector was introduced into rice protoplasts (Yu et al. 2014). A confocal laser-scanning microscope (Zeiss LSM 780, Germany) was used to study GFP fluorescence.

### Yeast Two-Hybrid Analysis, Pull-down, and Bimolecular Fluorescence Complementation (BiFC)

The *OsWHY1* coding sequence was cloned into pGBKT7 (BD), while *OsTRX* z was cloned into pGADT7 (AD). After that, the OsWHY1-BD and OsTRX z-AD plasmids were co-transformed into yeast cells (Y2H Gold strain) and incubated for 3 days at 30 ℃ on a solid medium without leucine and tryptophan. The colonies were combined in 50 ul of sterile water before plating on a solid medium containing X-α-Gal but without leucine, tryptophan, histidine, and adenine. The *OsWHY1* was cloned into the pET-28a vector to produce the His-OsWHY1 vector for pull-down assays. In addition, the coding sequence of *OsWHY1* was cloned into the 2YN vectors to construct nYFP-OsWHY1. OsTRXz-cYFP and GST-OsTRX z vectors were obtained from our previous studies (He et al. 2018). The pull-down and BiFC assays were performed according to our previously reported methods (He et al. 2018). Additional file 2: Table S2 contains a list of all primers used for interaction detection.

### Sequence and Phylogenetic Analysis

Gramene (http://www.gramene.org/) was used for gene prediction and structural analysis. Proteins homologous to OsWHY1 were identified by the National Center for Biotechnology Information (NCBI, http://www.ncbi.nlm.nih.gov/). Subcellular location prediction was performed using the plant proteome database (PPDB) (http://ppdb.tc.cornell.edu/dbsearch/searchacc.aspx). The DNAMAN program was used to achieve multiple sequence alignments. The MEGA v7.0 software was used to create a neighbor-joining with the bootstrap method and 1000 replicates.

## Supplementary Information

**Additional file 1.** Supplemental Figures.

**Additional file 2**. Supplemental Tables.

## Data Availability

All data supporting the conclusions of this article are provided within the article (and its additional files).

## Abbreviations

WHY: Whirly
Y2H: Yeast two-hybrid
BiFC: Bimolecular fluorescence complementation
CRISPR-Cas9: Clustered regularly interspaced short palindromic repeats–CRISPR-associated protein 9
Chl: Chlorophyll
CP: Chloroplast
Car: Carotenoids
GFP: Green fluorescent protein
CTP: Chloroplast transit peptide
qRT-PCR: Quantitative real-time polymerase chain reaction
TEM: Transmission electron microscopy
WT: Wild type
NEP: Nucleus-encoded RNA polymerase
PEP: Plastid-encoded RNA polymerase
TAC: Transcriptionally active chromosome
RGAP: Rice genome annotation project
NCBI: National center for biotechnology information
PPDB: The plant proteome database
