# Supplementary material for: OsWHY1 Interacts with OsTRX z and is Essential for Early Chloroplast Development in Rice
Source: Rice (N Y). 2022 Oct 8;15:50. doi: 10.1186/s12284-022-00596-y (PMC9547768; doi:10.1186/s12284-022-00596-y)
Supplement: Supplementary file 1 — Additional file 1. Supplemental Figures. [file 12284_2022_596_MOESM1_ESM.docx]

**Additional file 1: Supplemental Figures**

**
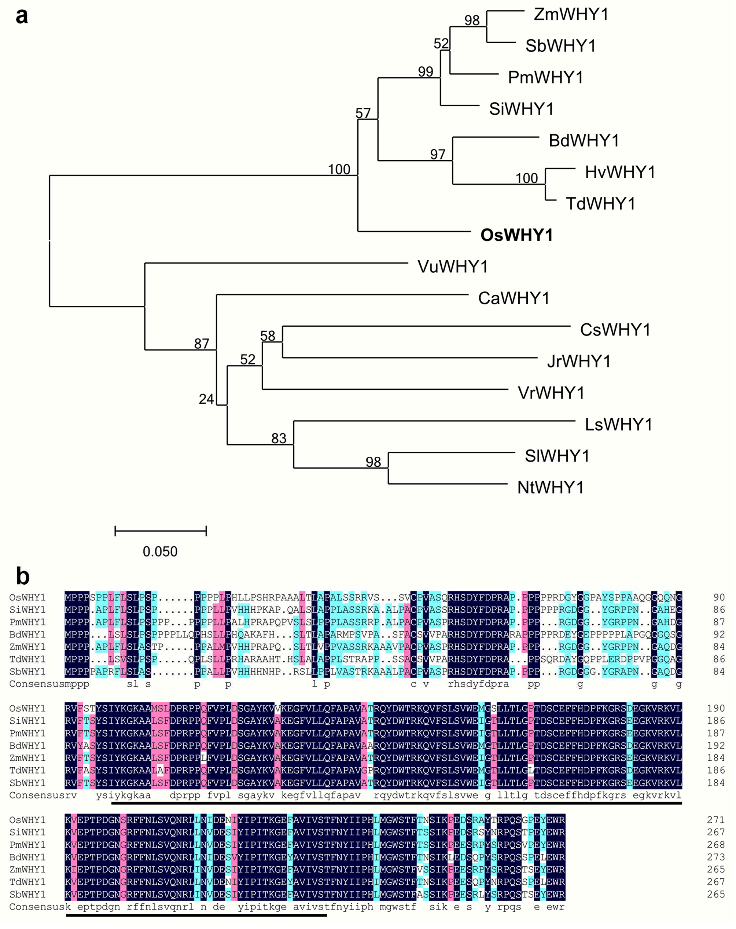
**

**Fig. S1** Phylogenetic analysis and amino acid sequence alignment

**a** The Neighbor-Joining method constructed the phylogenetic tree via MEGA v7.0. **b** OsWHY1 homolog amino acid sequence alignment. Amino acids that were fully or partially conserved are shaded blue and green, respectively. The Whirly domain is labeled with a black line under the sequences. Protein sequences are SiWHY1 (XP_004964537.1, *Setaria italica*), PmWHY1 (RLN12590.1, *Panicum miliaceum*), BdWHY1 (XP_003557198.1, *Brachypodium distachyon*), HvWHY1 (KAE8776928.1, *Hordeum vulgare*), ZmWHY1 (NP_001123589.1, *Zea mays*), TdWHY1 (XP_037459556.1, *Triticum dicoccoides*), SbWHY1 (XP_002436467.1, *Sorghum bicolor*), VuWHY1 (XP_027917372.1, *Vigna unguiculata*), VrWHY1 (XP_034694814.1, *Vitis riparia*), CaWHY1 (XP_027098346.1, *Coffea arabica*), SlWHY1 (NP_001289829.2, *Solanum lycopersicum*), CsWHY1 (XP_030484648.1, *Cannabis sativa*), LsWHY1 (XP_023742985.1, *Lactuca sativa*), JrWHY1 (XP_018812704.1, *Juglans regia*), NtWHY1 (XP_016474450.1, *Nicotiana tabacum*).

**
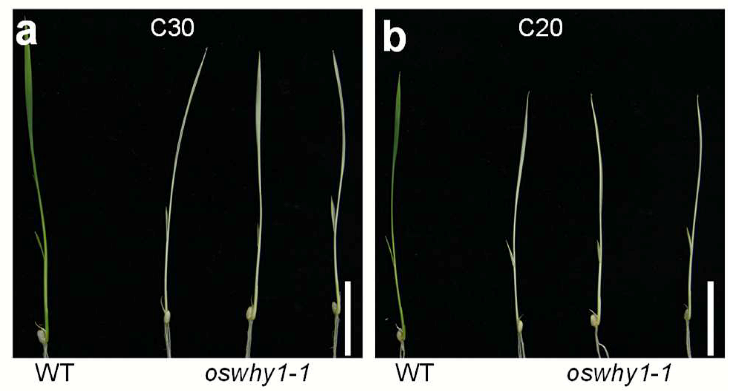
**

**Fig. S2** Phenotypes of WT and *oswhy1-1* plants under different temperature conditions.

**a** C30, 14 h : 10 h (light : dark) conditions at constant 30 ℃. **b** C20, 14 h : 10 h (light : dark) conditions at constant 20 ℃. Bar = 4 cm.


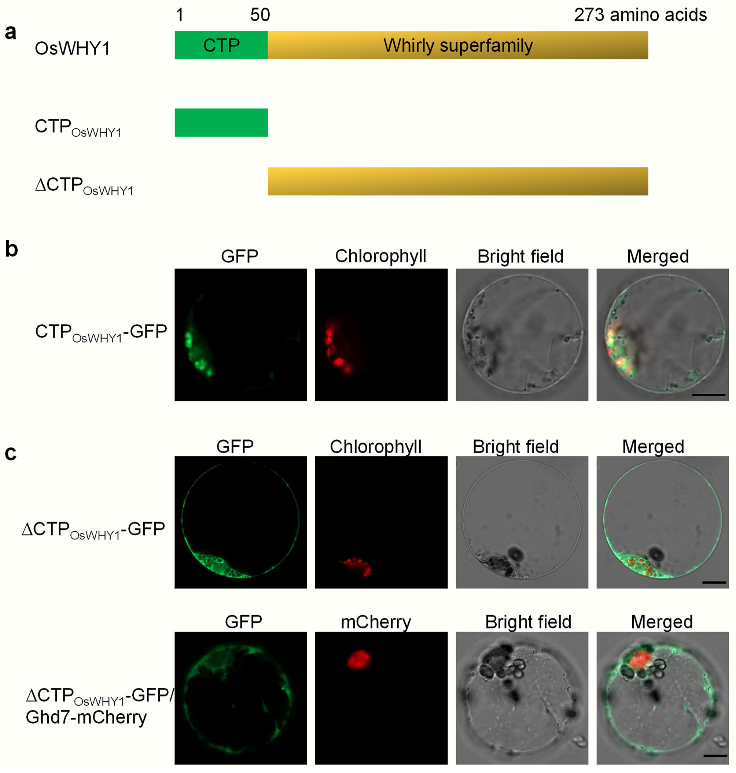


**Fig. S3** Subcellular localization of CTP_OsWHY1_-GFP and ΔCTP_OsWHY1_-GFP

**a** Diagrams of OsWHY1, CTP_OsWHY1_, and ΔCTP_OsWHY1_ protein sequences. 1-50 amino acids are the chloroplast transit peptide (CTP) sequences of OsWHY1. **b** The CTP_OsWHY1_-GFP fusion protein was localized in the chloroplast. **c** The CTP_OsWHY1_-GFP fusion protein was localized in the cytoplasm. The Ghd7-mCherry protein was a nuclear localization marker. Bar = 5 μm.
