# Supplementary material for: OsWHY1 Interacts with OsTRX z and is Essential for Early Chloroplast Development in Rice
Source: Rice (N Y). 2022 Oct 8;15:50. doi: 10.1186/s12284-022-00596-y (PMC9547768; doi:10.1186/s12284-022-00596-y)
Supplement: Supplementary file 2 — Additional file 2. Supplemental Tables. [file 12284_2022_596_MOESM2_ESM.docx]

**Additional file 2: Supplemental Tables**

**Table S1** Analysis of chloroplast RNA editing (C to U) in the WT and *oswhy1-1* mutant

| Gene | Editing position | Edited codon | Amino acid change | Transfer C to U (Y/N) | |
| --- | --- | --- | --- | --- | --- |
|  |  |  |  | WT | *oswhy1-1* |
| *atpA* | C1148 | uCa | S→L | Y | Y |
| *ndhA* | C473 | uCa | S→L | Y | Y |
|  | C563 | uCa | S→L | Y | Y |
|  | C1070 | uCc | S→F | Y | N |
| *ndhB* | C467 | cCa | P→L | Y | Y |
|  | C586 | Cau | H→Y | Y | Y |
|  | C611 | uCa | S→L | Y | Y |
|  | C704 | uCc | S→F | Y | Y |
|  | C737 | cCa | P→L | Y | Y |
|  | C830 | uCa | S→L | Y | Y |
|  | C836 | uCa | S→L | Y | Y |
|  | C1481 | cCa | P→L | Y | Y |
| *ndhD* | C878 | uCa | S→L | Y | Y |
| *ndhF* | C62 | uCa | S→L | Y | Y |
| *ndhG* | C-10 | 5’-UTR | C→U | Y | Y |
|  | C347 | cCa | P→L | Y | Y |
| *rpl2* | C2 | aCg | T→M | Y | Y |
| *rpoB* | C467 | uCg | S→L | Y | Y |
|  | C545 | uCa | S→L | Y | Y |
|  | C560 | uCa | S→L | Y | Y |
| *rps8* | C182 | uCa | S→L | Y | Y |
| *rps14* | C80 | uCa | S→L | Y | N |
| *ycf3* | C185 | aCg | T→M | Y | Y |

**Table S2** List of primer pairs used in this study

| **RNA splicing test primer** | | | | |
| --- | --- | --- | --- | --- |
| Gene | | Forward Sequence (5’-3’) | Reverse sequence(5’-3’) | |
| *atpF* | | ATGAAAAATGTAACCCATTCTT | TTCATCGCCCTTTGTTTTTC | |
| *ndhA* | | ATGATAATAGACAGGGTACAGG | TTATAGTGAAACAAGTTGGGAAG | |
| *ndhB* | | ATGATCTGGCATGTACAGAATG | CTAAAAGAGGGTATCCTGAGCA | |
| *petB* | | TTCTCATATACGGTTCTCGG | TAAAGGGCCCGAAATACCTT | |
| *petD* | | ATGGGAGTAACAAAGAAACC | TGTTGCTCCAATACCTAACC | |
| *rpl2* | | ACGGCGAAACATTTATACAA | TTACTTACGGCGACGAAGAATA | |
| *rpl16* | | ATGCTTAGTCCCAAAAGAAC | AACCGAAGAAATTGACTTCG | |
| *rps16* | | AAAACGATGTGGTAGAAAGC | AGAATTCCGCCTTCCTTAAA | |
| *trnA* | | GGGGATATAGCTCAGTTGGT | TGGAGATAAGCGGACTCGAA | |
| *trnG* | | TCGTTAGCTTGGAAGGCTAG | GCGGGTATAGTTTAGTGGTA | |
| *trnI* | | TGGGCCATCCTGGACTTGA | AGCTCAGTGGTAGAGCGCG | |
| *trnK* | | GGTTGCCCGGGACTCGAA | GGGTTGCTAACTCAATGGTAGAG | |
| *trnL* | | GGATATGGCGAAATCGGTA | TGGGGATAGAGGGACTTGA | |
| *trnV* | | TAGGGCTATACGGATTCGAA | AGGGCTATAGCTCAGTTCGG | |
| *ycf3* | | ATGCCTAGATCCCGTATAAATG | TTATTCAAATTCAAAGCGCTTC | |
| *rps12* | | ACTATCAACCCCAAAAAACC | TTTGGCTTTTTGACCCCAT | |
| *23S* | | TTCAAAAGAGGAAAGGCTTG | AGAGAGCACTCATCTTGGGG | |
| **RNA editing test primer** | | | | |
| Gene | | Forward Sequence(5’-3’) | | Reverse sequence (5’-3’) |
| *ndh*A-1 | | GGACCGTCTATAGCAGTCAT | | CTGACGCCAAAGATTCCATC |
| *ndh*A-2 | | GGTGGAATTTGTCTATTCCC | | TTTCTCTTGTTTGAGAGGAC |
| *ndh*B-1 | | ATGATCTGGCATGTACAGAATG | | CTAAAAGAGGGTATCCTGAGCA |
| *ndh*B-2 | | CTTGGTTTCAATAGGACTCC | | TAAAAGAGGGTATCCTGAGC |
| *ndh*D | | ATTTTGGCTTCCTTATTGC | | GCCTCTACCCTGTCAACG |
| *ndh*F | | ATATGCATGGGTAATCCCTC | | AGTGGCTCCTAAGAAAAGTG |
| *ndh*G-1 | | CCTAATCCCTTTTTTCTTCC | | TCAAGACATTTATAGCTCCC |
| *ndhG-2* | | ATGGATTTACCTGGGCCAAT | | TTATTGCCGAGCCATAGTAA |
| *rpl*2 | | CCGGGTTATTCTATTCCACT | | TACGCATTTCGATTAGGGTC |
| *rps*8 | | ATGGGCAAGGACACTATTG | | AАСAТAAGACTTCTCCCCCA |
| *rps*14 | | ATGGCAAAAAAAAGTTTGATTC | | TTACCAACTGGATCTTGTTGCA |
| *rpo*B | | GTCCTGSDGTATTTACTACCGC | | TCCCCACCTACACAAGCAAA |
| *atp*A | | CCCAGGGGATGTTTTTTATT | | TGAAAAAAGCGTCCATTGTG |
| *ycf*3 | | TGTGGTAAGAAGGGGTTTCG | | GCGAATAATTCCGACAACCT |
| *rpo*C2 | | GGTCCTTGGGGATTCTTGAT | | TCTTGTTTTGTGGGTAACGG |
| **Plasmid construction** | | | | |
| Gene | Forward Sequence(5’-3’) | | | Reverse sequence (5’-3’) |
| OsWHY1-GFP | caggagctcggtaccggatccATGCCGCCACCGTCGCCG | | | gcccttgctcaccatggatccCCTCCGCCATTCATATTCAGG |
| nYFP-OsWHY1 | aattaaggcgcgccactagtATGCCGCCACCGTCGCCG | | | ctgccacctcctccactagtCCTCCGCCATTCATATTCAGG |
| His-OsWHY1 | agcaaatgggtcgcggatccATGCCGCCACCGTCGCCG | | | cggagctcgaattcggatccCCTCCGCCATTCATATTCAGG |
| GST-TRXz | gatctggttccgcgtggatccATGGCCATGGCCGCGGCC | | | cccgggaattccggggatccTCACAATTCATTATCAATGATATTTCTGA |
| OsWHY1-AD | gaggccagtgaattcATGCCGCCACCGTCGCCG | | | acccgggtggaattcTCACCTCCGCCATTCATATTCA |
| OsTRX z-BD | atggaggccgaattcATGGCCATGGCCGCGGCC | | | gatccccgggaattcTCACAATTCATTATCAATGATATTTCTGA |
| CTP_OsWHY1_-GFP | caggagctcggtaccggatccATGCCGCCACCGTCGCCG | | | gcccttgctcaccatggatccCACGGGGCAGACGGAAGA |
| ΔCTP_OsWHY1_-GFP | caggagctcggtaccggatccATGGCCTCCCAGCGCCAC | | | gcccttgctcaccatggatccCCTCCGCCATTCATATTCAGG |
| **qRT-PCR** | | | | |
| Gene | Forward Sequence(5’-3’) | | | Reverse sequence (5’-3’) |
| *OsWHY1* | GGGATTCGTGCTGCTACAGT | | | GCTACCCATCTCCCACACAG |
| *OsTRX z* | TGTGAAGGTGGATACTGATGATGA | | | GTCTTTGCTTTGATCTGGACTGAA |
| *psaA* | GTTTTCGCGGAGGGCTAGAT | | | TGACCTGCGATCAGGAAAAGA |
| *psbA* | ACTAGCACCGAAAACCGTCTTT | | | CAGCGATGAAGGCGATAATAAA |
| *rbcL* | CTCGCGGTATCTTTTTCACTCA | | | TCGGTCAGAGCTGGCATATG |
| *rpoB* | CAAGTTTTCGGAGCCGAGAT | | | GCTAAAGATCCAGTAAGTCCAACGT |
| *rpoC1* | TCCGTCGGAACAACAATCTTG | | | TCCACGGCTTCTTGTACCAAT |
| *rpoC2* | ATGCATCGCAGGTACACCAA | | | CCCTCGCGTAAATTGCTTTG |
| *HEMA1* | GAACTCACCAGTCTGAATCATATTGA | | | CATCCAGTCTACCACTTCTCTAATCC |
| *HEMC* | TGCTTGACTGCAAGTTCCCTTG | | | CTAGAGCCAACAATGTAGCATGG |
| *HEME* | AGGAGCAGGTGAGGGAGCT | | | TGGCGTCTGCAAGGTGAGAC |
| *CHLI* | GTTCGAGCCTGGTTTGCTTGC | | | CTCTCCACGGTGTTCCATCCTG |
| *CHLD* | GCTTGCAGAAAGCTACACAAGC | | | AGGCCGTGAGCTAAAGGAGA |
| *CHLM* | CCATCCATTGGTCTCCTTATGACA | | | GTAGCCTACTTACCATCAATGAGTC |
| *PORA* | TGTACTGGAGCTGGAACAACAACT | | | TCAATAGCACATCACTCTCACTCACT |
| *DVR* | CAGGTCGAGACCGTCAAGAAC | | | ATGACCTGGATCGGCACCTTG |
| *YGL1* | GATAGAGCTCTGGGGCTTCAGTC | | | GCTTGCCGGAACTGAAAAGGTAG |
| *CAO1* | GACACCTTCATCTGGGCTTCAA | | | CGAGAGACATCCGGTAGAGC |
| *16S rRNA* | CCGTTGGTGTTCTTTCCGAT | | | TTCAAGTCCGCCGTCAAATC |
| *23S rRNA* | TGTGGGCGTTAGAGCATTGAG | | | CACTTGGCTACCCAGCGTTTA |
| *rps4* | CGATTAGGTATGGCTTCAAC | | | GATCTTTGGTTATCCTTCGTAG |
| *rps11* | GGTGCTGGTAGTGGAAGAGAT | | | GCGGCATAGGTGTTACATCG |
| *rps12* | AGCCGTTTGCTACCAATGG | | | TGATCGGTACCAATGAATAGG |
| *rps14* | GTTTGATTCAGAGAGAGAGG | | | TTCTCGAAGTATGTGTCCGG |
| *rps16* | CCTCGCGACAGACGTCCTAT | | | CTCCTCGTTAGGTGCTCCATC |
| *rps18* | CAACCTTTTCGCAAACCCAA | | | ATAATCAATTCGATCCCCCG |
| *rps19* | CGGGCATCTAGCATTCTACC | | | CCCAATTTGCGACCTACCATA |
| *rpl16* | ATGCTTAGTCCCAAAAGAACCAG | | | GCCTCGCCGTAATCCAAGT |
| *rpl20* | AGGTAAGAAGAGCGTTTGTTTCC | | | TGCTGCGTTTATCCGACTGA |
| *UBQ5* | CTCGCCGACTACAACATCCA | | | TCTTGGGCTTGGTGTACGTCTT |
